# Increasingly detailed insights in animal behaviours using continuous on-board processing of accelerometer data

**DOI:** 10.1186/s40462-022-00341-6

**Published:** 2022-10-24

**Authors:** Hui Yu, Chris A.J. Klaassen, Jian Deng, Trent Leen, Guozheng Li, Marcel Klaassen

**Affiliations:** 1 Centre for Integrative Ecology, School of Life and Environmental Sciences, Deakin University, Geelong, VIC Australia; 2 Druid Technology Co., Ltd, Chengdu, Sichuan China; 3 Experimental Zoology Group, Wageningen University, Wageningen, the Netherlands; 4 Korteweg-de Vries Institute for Mathematics, University of Amsterdam, Amsterdam, the Netherlands; 5 Geelong Field & Game, Balliang East, VIC Australia; 6 Wetlands Environmental Taskforce, Seymour, VIC Australia

**Keywords:** Wildlife tracking, Machine learning, Time-activity budget, Daily distance, Home range

## Abstract

**Background:**

Studies of animal behaviour, ecology and physiology are continuously benefitting from progressing biologging techniques, including the collection of accelerometer data to infer animal behaviours and energy expenditure. In one of the most recent technological advances in this space, on-board processing of raw accelerometer data into animal behaviours proves highly energy-, weight- and cost-efficient allowing for continuous behavioural data collection in addition to regular positional data in a wide range of animal tracking studies.

**Methods:**

We implemented this latest development in collecting continuous behaviour records from 6 Pacific Black Ducks *Anas superciliosa* to evaluate some of this novel technique’s potential advantages over tracking studies lacking behavioural data or recording accelerometer data intermittently only. We (i) compared the discrepancy of time-activity budgets between continuous records and behaviours sampled with different intervals, (ii) compared total daily distance flown using hourly GPS fixes with and without additional behavioural data and (iii) explored how behaviour records can provide additional insights for animal home range studies.

**Results:**

Using a total of 690 days of behaviour records across six individual ducks distinguishing eight different behaviours, we illustrated the improvement that is obtained in time-activity budget accuracy if continuous rather than interval-sampled accelerometer data is used. Notably, for rare behaviours such as flying and running, error ratios > 1 were common when sampling intervals exceeded 10 min. Using 72 days of hourly GPS fixes in combination with continuous behaviour records over the same period in one individual duck, we showed behaviour-based daily distance estimation is significantly higher (up to 540%) than the distance calculated from hourly sampled GPS fixes. Also, with the same 72 days of data for one individual duck, we showed how this individual used specific sites within its entire home range to satisfy specific needs (e.g. roosting and foraging).

**Conclusion:**

We showed that by using trackers allowing for continuous recording of animal behaviour, substantial improvements in the estimation of time-activity budgets and daily traveling distances can be made. With integrating behaviour into home-range estimation we also highlight that this novel tracking technique may not only improve estimations but also open new avenues in animal behaviour research, importantly improving our knowledge of an animal’s state while it is roaming the landscape.

**Supplementary Information:**

The online version contains supplementary material available at 10.1186/s40462-022-00341-6.

## Introduction

Many fields within animal biology and notably studies in animal behaviour and movement ecology have benefited greatly from the development of advanced biologging techniques [1–3]. Nowadays, tags can not only log spatiotemporal information of animals, but also record animal physiology, behaviour and ambient environmental information by various on-board miniature sensors [4]. Among the sensors, accelerometer (ACC) data has been mainly used to study animal behaviours and energy expenditure [e.g. 5]. However, large amounts of raw ACC data can be a burden to limited on-board storage or device battery capacity when data have to be transmitted remotely [6].

On-board data processing, to shrink raw data volume, is one way to solve the constraints in remote behavioural data collection and transmission using ACC data. Popular ACC data processing procedures, be it used on-board of tracking devices or during post-processing, involve the calculation of activity indices over pre-defined time windows of ACC data such as ODBA (Overall Dynamic Body Acceleration, e.g. [7]), VeDBA (Vectorial Dynamic Body Acceleration, e.g. [8]) and RMS (Root Mean Square, e.g. [9]). These indices have been mostly used to represent activity levels of tracked animals (e.g. RMS; [10]) or to study animals’ energy expenditure (e.g. VeDBA; [11]). When these indices were calculated on-board of trackers, the research period could be extended considerably, even when using small trackers on small animals. As a prime example, Bäckman, Andersson [12] used data loggers that summarized single axis ACC data into 12 activity indices (by scoring relative intensity of ACC data) per hour to study migration and activity patterns of the red-backed shrike *Lanius collurio* for up to 14.5 months. Similar data processing procedures have also been used in biomechanics studies using ACC data. For example, indices such as ODBA have been used to evaluate fish swimming behaviour and effort [13]. Also, tail beat frequencies derived from ACC data were used to approximate swimming speed in sailfish *Istiophorus platypterus* [14]. Yet other on-board ACC data processing focussed on the animal’s biomechanics were used by Cox, Orgeret [15], summarizing ACC data on-board of satellite transmitters to study swimming effort and pitch angle (body angle relative to the horizontal plane) of southern elephant seals *Mirounga leonine*.

Accelerometers have also been widely used to study animal behaviour. ACC data is translated into behaviour using either unsupervised (e.g. [16]) or supervised (e.g. [17]) machine learning. Supervised behaviour classification relies on direct behavioural observations to assist in the translation process of the ACC data, whereas unsupervised behaviour classification relies on the ACC data itself and post-hoc behavioural interpretation of the ACC classes. Many supervised behaviour classification studies make use of machine learning where a range of summary statistics or “features” are calculated from the segmented ACC data. Next, using segments for which also direct behavioural observations are available, a behaviour classification model is “trained” using machine learning. In unsupervised behaviour classification studies, ACC data is clustered based on similarities in the ACC data after which expert opinion is used to allocate a likely behaviour to each cluster. For instance, unsupervised behaviour classification consists of processing ACC data by wavelet transformation followed by k-means clustering (e.g. [18]). Several studies applied on-board ACC data processing for animal behaviours and demonstrated their advantages. Nuijten et al. (2020) calculated raw ACC data into features on-board of trackers to study behaviours of Bewick’s swans *Cygnus columbianus bewickii*. In this way, they were able to sample one, 2 s bout of ACC data every 2 min instead of sampling one bout of raw ACC data every 15 min. They found that rare behaviours were picked up significantly more frequently when using on-board data processing. Korpela et al. (2020) used on-board behaviour classification through ACC data to control data sampling of the more energy consuming on-board camera to extend the runtimes of field experiments. Recently, we developed a tracking system [19] using on-board continuous behaviour classification from ACC data that proved to be energy-, weight- and cost-efficient and allowed for continuous recording of behaviour that could be transmitted through the 3G mobile network on a daily basis.

Our previous work [19] focused on describing the functioning and technical performance of the new tracking system using on-board and continuous behaviour classification in six free-ranging Pacific black ducks *Anas superciliosa*. Here, using the same data set, we focus on how this system can enhance our ecological understanding of animals when compared to systems that lack this functionality (i.e. GPS tracker without accelerometer) or only record ACC data intermittently. The trackers used continuously recorded eight different behaviours complemented with hourly GPS positions. Aside from providing details of the continuously recorded behaviour for these six individuals, we provide in-depth analyses covering 72 days of continuous behavioural and hourly positional data for one individual. To illustrate how continuous on-board classification of animal behaviours can benefit animal-behaviour and movement-ecological studies, we used the data thus obtained to compare time-activity budgets using continuous versus intermittently sampled ACC data. We also compared energetically costly displacement behaviour estimates calculated from continuous behaviour records with calculations based on hourly positional data in isolation. Finally, we used the behaviour records for a more in-depth home range estimation, allowing focussing on which parts of their environment animals display specific (critical) behaviours and how their timing may vary on a daily and seasonal basis.

Home range is a concept in animal ecology that has found wide use since the beginnings of animal telemetry [20]. In most studies, home range is typically estimated based on animals’ geographical locations over time. Although these methods estimate the geographical distribution of animals and how that may vary over time, they may not be able to inform on how and why animals use their home range to satisfy their (daily, annual and life cycle) needs. Instead of using time spent in spatial locations for home range description, Powell and Mitchell [21] proposed that more metrics can be used to better describe animals’ home range. For this they suggested a range of potential metrics such as energy expenditure, energy gained, giving-up densities for resources, danger from predators, potential for competition, potential for mutualisms and access to mates. Following some of the suggestions of Powell and Mitchell [21] to better assess the environmental requirements of animals, we calculated and depicted an animal’s home range integrating not only spatial and temporal information from GPS fixes, but also using energy expenditure and detailed behaviour information from ACC data. Aside from illustrating these advancements, we also discuss additional ways in which on-board data processing of ACC data can assist in studying and conserving free-ranging animals in their natural environments.

## Methods

### Duck tracking

Details on the properties and functioning of the GPS-3G-Bluetooth trackers, including the on-board ACC data processing method and performance, as well as the deployment of the trackers on the six Pacific Black Ducks can be found in Yu, Deng [19]. In brief, the trackers logged GPS fixes at hourly intervals when battery levels allowed (i.e. >= 3.7v). The on-board accelerometer was configured at always-on mode, sampling tri-axial ACC data at 25 Hz. Every 10 min, tri-axial ACC data was summarised into one mean overall dynamic body acceleration value (ODBA). Every 2 s, ACC data was processed into 1 behaviour code out of 8 alternative behaviour types, namely dabbling, feeding, floating, flying, preening, resting, running and walking. The recorded GPS fixes, ODBA values and behaviour codes were scheduled to be transmitted through the 3G mobile network on a daily basis and stored on board for later transmission in case battery power was insufficient for transmission. Six Pacific Black Ducks *Anas superciliosa* were tracked, i.e. d5099_2 (3/2/2021-30/3/2021), d5241 (4/1/2021- 13/3/2021), d5239 (23/12/2020-17/2/2021), d5246 (3/2/2021-13/4/2021), d5248 (24/11/2020-21/1/2022) and d5210 (20/11/2020-4/10/2021).

### Evaluation time-activity budget accuracy when sampling ACC data in bursts

We used the continuous behaviour records from the six ducks to evaluate time-activity budget accuracy using a range of ACC sampling intervals (i.e. every 10 s, 30 s, 1 min, 3 min, 5 min, 10 min, 20 min, 30 and 60 min). We only used data from days where the behaviour data covered at least 99.5% of the day or > 43,000 behavioural records. We also discarded the first and the last day of tracking of each individual, leaving a total of 690 days of continuous behaviour records. With each sampling interval, we chose the first behaviour of the day as the starting point for sampling and sampled behaviours with an increment equal to the sampling interval until the end of the day. For every day of recording and each behaviour the difference was calculated between the proportion of time (%) it was observed using the continuous recordings minus the sampled recordings. Standard deviations of these daily differences per behaviour (*sd_sampling*) were calculated across all 690 days for each sampling interval. Daily mean proportion per behaviour (*p*) was calculated using all continuous behaviour records (i.e. one *p* value per behaviour). Error ratio per sampling interval per behaviour was calculated using equation:

1 $${\rm{error}}\,{\rm{rati}}{{\rm{o}}_{{\rm{ij}}}}{\rm{ = }}\frac{{{\rm{sd\_samplin}}{{\rm{g}}_{{\rm{ij}}}}}}{{{{\rm{p}}_{\rm{j}}}}},$$

where *i* represents different sampling intervals and *j* represents different behaviours. To evaluate how this error ratio varies between common and rare behaviours and depends on sampling interval, we calculated a multiple linear regression using the natural logarithm of the error ratio as dependent variable and the natural logarithm of sampling interval (in minutes) and the natural logarithm of *p* (%) as explanatory variables.

As shown in the supplemental material (supplementary Eq. 2.4), if we assume the sampled behaviour sequences to consist of independent observations, the expected error ratio equates to:

2 $${\rm{E\_error}}\,{\rm{rati}}{{\rm{o}}_{{\rm{ij}}}} = \frac{{\sqrt {\frac{{{p_j}(1 - {p_j})}}{{{n_i}}}} }}{{{p_j}}},$$

where *n*_*i*_ is the number of records in each day for a specific sampling interval *i*.

Notably for behaviour sequences collected at small sampling intervals, the assumption of independence is likely to be violated. In supplemental material we provide a measure of dependence for specific behaviours, which varies between 0 (no dependence) and 1 (full dependence (see supplementary Eq. 4.12):

3 $$Dependence= \frac{{\sum }_{i=1}^{k}|{q}_{i}-{p}_{i}|}{2(1 - {p}_{min})}$$

where *q*_*i*_ is the proportion of behaviour transition from the focal behaviour to all behaviours (i.e. *k* = 8), and *p*_*i*_ is the daily mean proportion of each behaviour.

For each of the eight behaviours we used this equation to calculate how dependence varied with sampling interval.

### Evaluating daily distance travelled

The distance travelled by animals is usually calculated by summing straight-line distances between adjacent GPS locations [22]. The flight bouts extracted from continuous behaviour records of ducks provide another way to estimate travel distance (we did not consider correcting travel distance by walking or running as most distance was covered by flight). We used 72 days of data (from 21/11/2020 to 31/01/2021) of one duck (d5210) to illustrate how integration of behavioural data can improve travel distance estimation. Excluding four days with incomplete GPS tracking, i.e. 15/01/2021, 16/01/2021, 22/01/2021, 29/01/2021, 68 days’ worth of data were retained for this exercise. Daily flight distance was calculated by summing Haversine distances between adjacent fixes [23] within each day. For the alternative calculation of flight distance using behavioural data, we only used flight bouts of at least 6 s duration to reduce potential noise from occasional wing flapping while on land or water. Daily travel distance was calculated by multiplying daily total time in flight by the mean flight speed, where we assumed a mean fight speed of 15 m/s based on the median flight speed (15.47 m/s) of a similar species - mallard *Anas platyrhynchos -* during non-migratory flight [24].

### Behaviour and energy-expenditure based home ranges

We used the same 72 days of data of d5210 as mentioned in the previous section to evaluate the bird’s home range integrating energy expenditure and continuous behaviour records. To this end we rasterized the map of the region used by duck 5210 during this 72-day period in 30 ⋅ 30 m grid cells. Each ODBA value and behaviour record was geographically assigned by the nearest GPS fix in time. To represent the duck’s energy use in each cell, the sum of the bird’s ODBA divided by the total ODBA (i.e. energy percentage) over the 72-day period was calculated for each grid cell. For behavioural analyses we selected grid cells where the duck spent at least a total of 24 h over the total 72-day period. For each of the thus selected 18 cells, the total relative time allocation to each of the 8 behaviours was calculated. We used hierarchical cluster analysis (function “hclust” in R) to cluster cells based on the similarity in behaviour expressed in each cell. We similarly clustered cells based on the time-of-day cells were used, using whole hour intervals. Finally, we calculated the total amount of time spent in each cell on each day within the 72-day focal period.

## Results

**Fig. 1:**
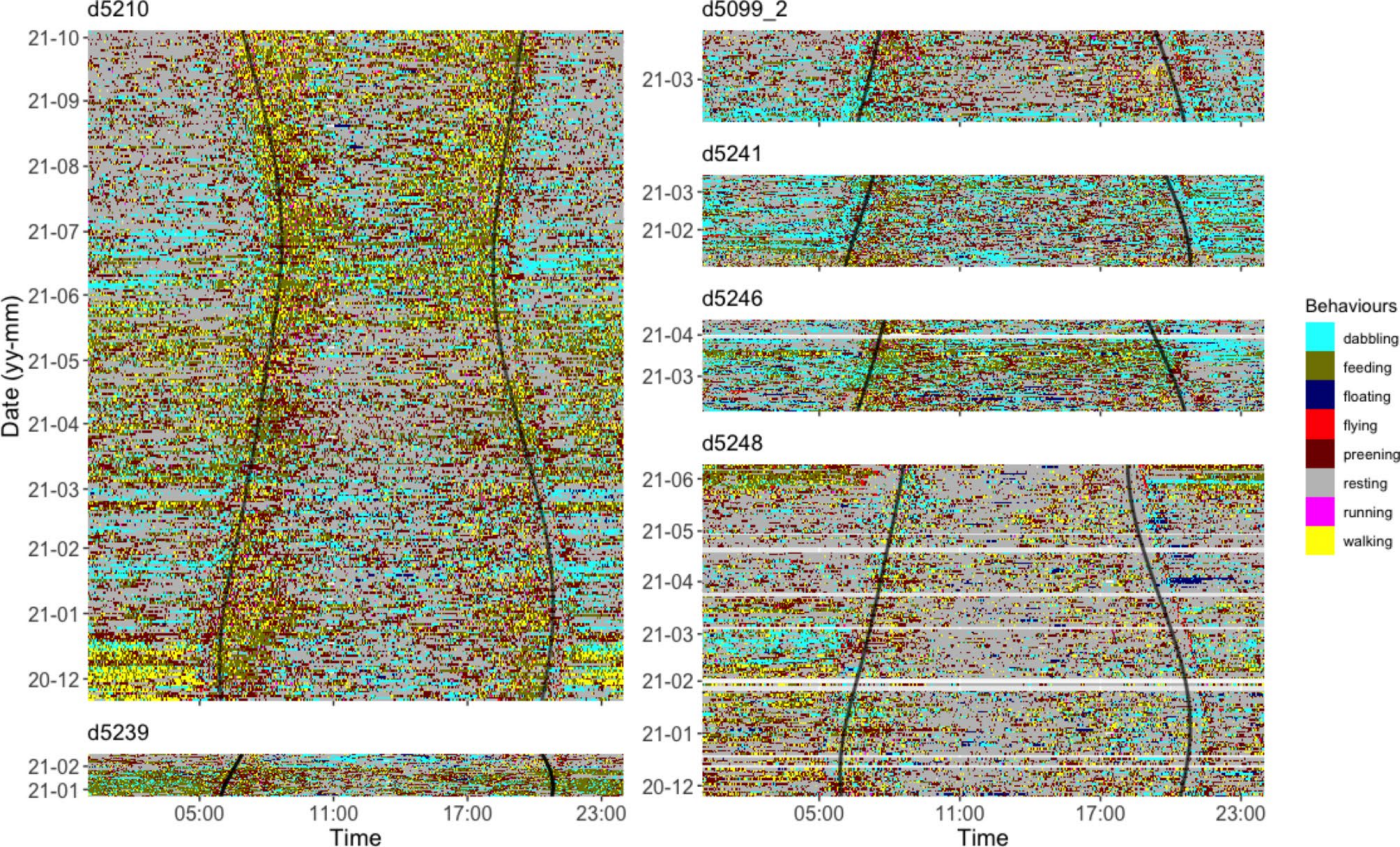
Continuous ethograms of six Pacific black ducks with date on the y-axis and time of day on the x-axis. Grey curves depict local sunrise and sunset times. For this representation, only the behaviour during the first 2 s of each half minute is used, the remaining 28 s being discarded

**Fig. 2:**
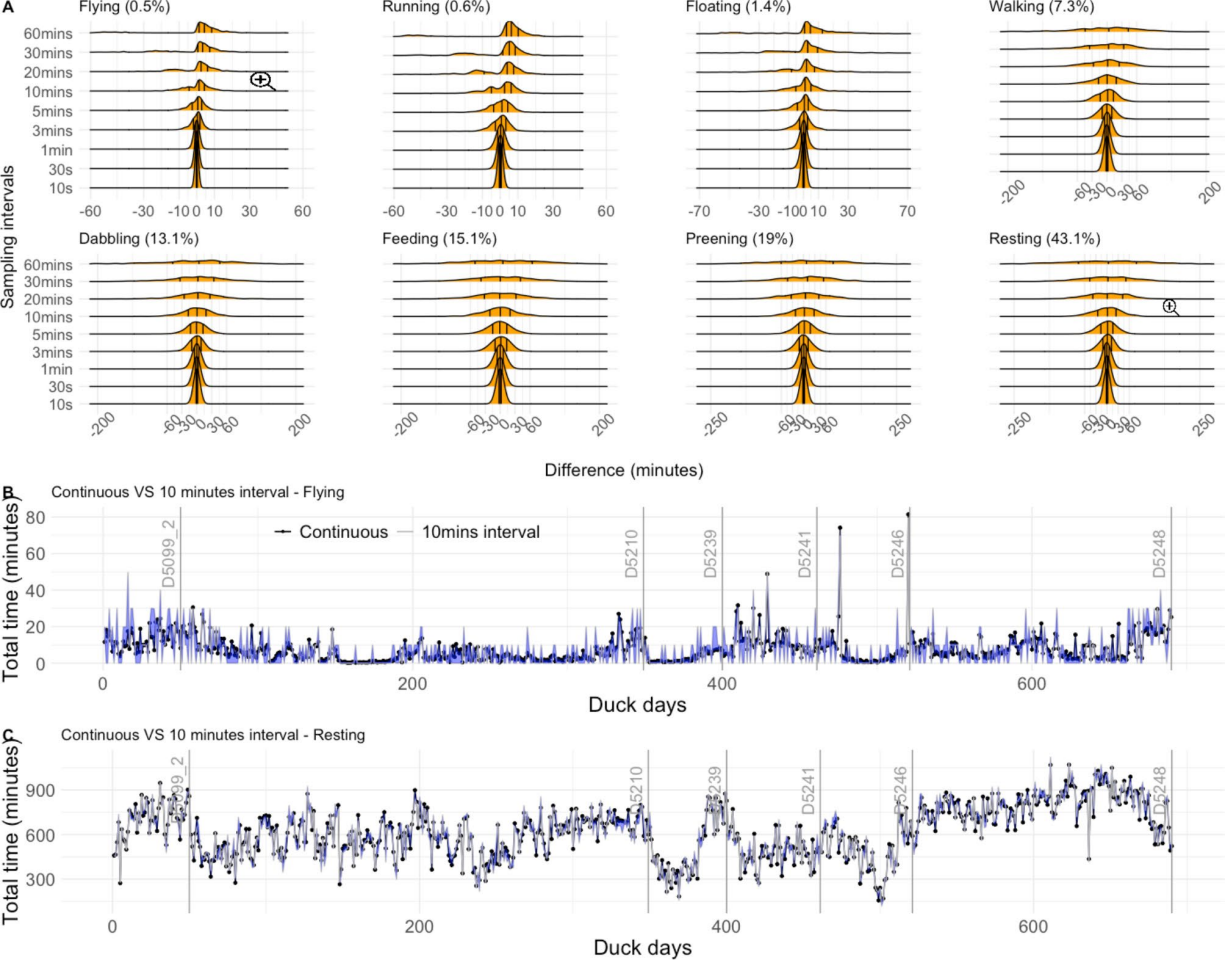
Time-activity budget estimation differences between down sampled behaviour records and continuous behaviour records (using a total of 690 days of continuous behaviour records across 6 ducks). (A) Density plots of differences for 9 sampling intervals varying from 10s to 60 min of 8 behaviour types ranked by their overall percentages. The three vertical lines in each density plot represent 25%, 50%, and 75% percentiles. (B) Daily total time in the rarest behaviour recorded: flying. For each duck day the total time spent flying was calculated from continuous behaviour records (marked with black dots) and data down sampled with 10 min interval (grey line without dots). The blue shades indicate the difference between the two calculations. Vertical grey lines indicate the separations between ducks (e.g. data between the line marked with D5099_2 and the line marked with D5210 is the data of D5210). (C) As B but for the most frequently recorded resting behaviour

**Fig. 3:**
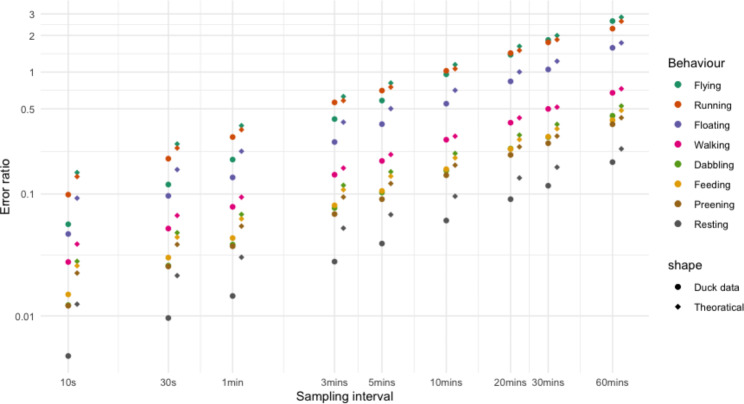
Using the same data as Fig. 1, error ratio (i.e. uncertainty of time-activity budget estimation through permutations with 9 sampling intervals: 10 s, 30 s, 1 min, 3 min, 5 min, 10 min, 20 min, 30 and 60 min) as a function of sampling interval and daily mean behaviour proportion (*p*) for the eight different behaviours. Colored dots indicate values out of permutations and colored diamonds indicate values calculated using Eq. 2

**Fig. 4:**
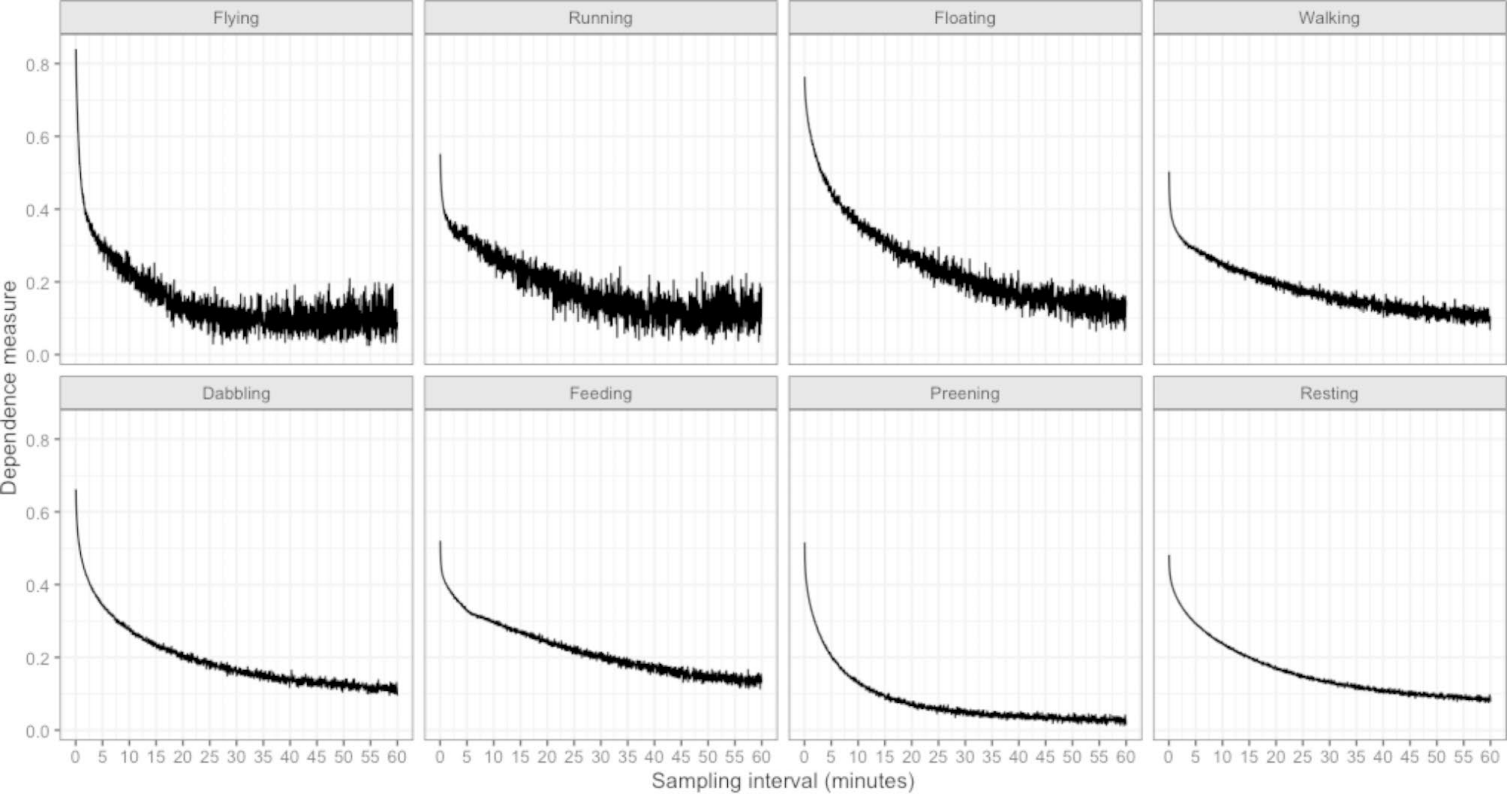
Measures of dependency between behavioural observations (cf. Eq. 3) as a function of sampling interval for all eight behaviours that were distinguished across the 690 days in the six ducks. Dependency was calculated using Eq. 3

Using 690 days of data from 6 ducks (Fig. 1), we illustrate that, as expected, uncertainty in time-activity budget estimation in each behaviour increased with an increase in sampling interval (Fig. 2). Moreover, the error ratio of the various behaviours not only increased with sampling interval, but also depended on the amount of time the animals typically engaged in a certain behaviour. A significant regression equation was found illustrating this (F(2, 69) = 2073, P < .001, R^2^ of 0.983) and showing that error ratio was highest in rare behaviours (Fig. 3; ln[error ratio] = -4.398 ± 0.05 (SE) + 0.588 ± 0.012 ln[sampling interval] – 0.58 ± 0.014 ln[*p*]). The expected error ratios assuming independence of observations (Eq. 2) were similar to those observed in the empirical duck dataset, notably for longer interval lengths (Fig. 3). This is likely due to dependency in behavioural observations being much higher at short compared to long intervals in our duck data (Fig. 4). Using Eq. (2), we calculated a set of error ratios using a range of proportions (i.e. 0.005, 0.01, 0.02, 0.05, 0.1, 0.2, 0.3, 0.4, 0.5) and sampling intervals (10s, 30s, 1 min, 3 min, 5 min, 10 min, 20 min, 30 min, 60 min) and presented these in Table 1.

**Table 1:**
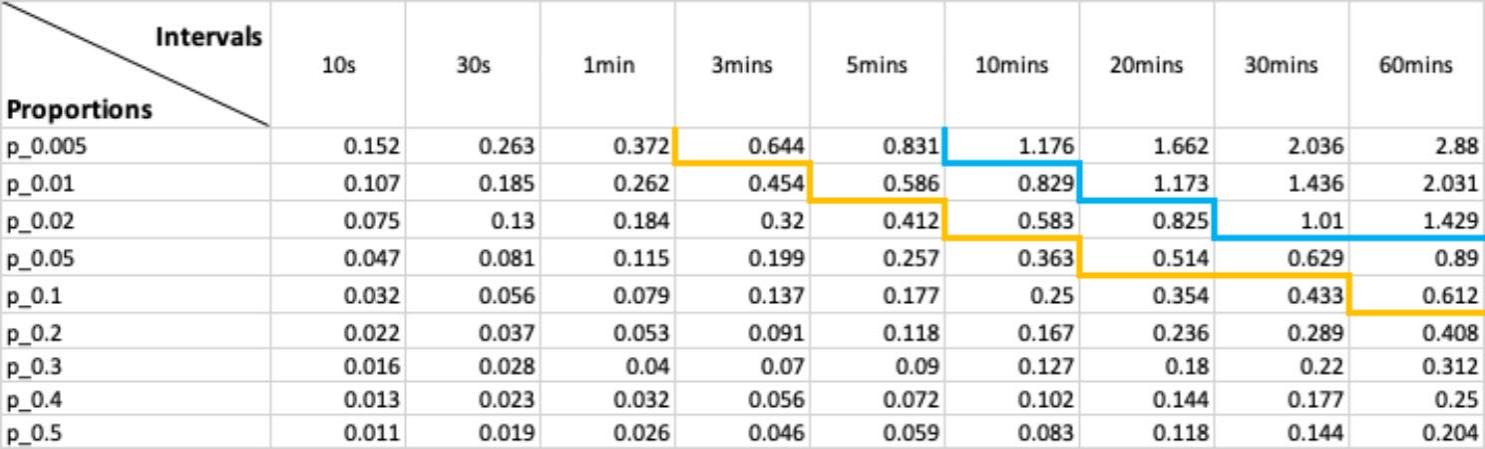
Error ratios calculated with different proportions and intervals. The orange curve indicates the border of error ratio < 0.5 and > 0.5. The blue curve indicates the border of error ratio < 1 and > 1

**Fig. 5:**
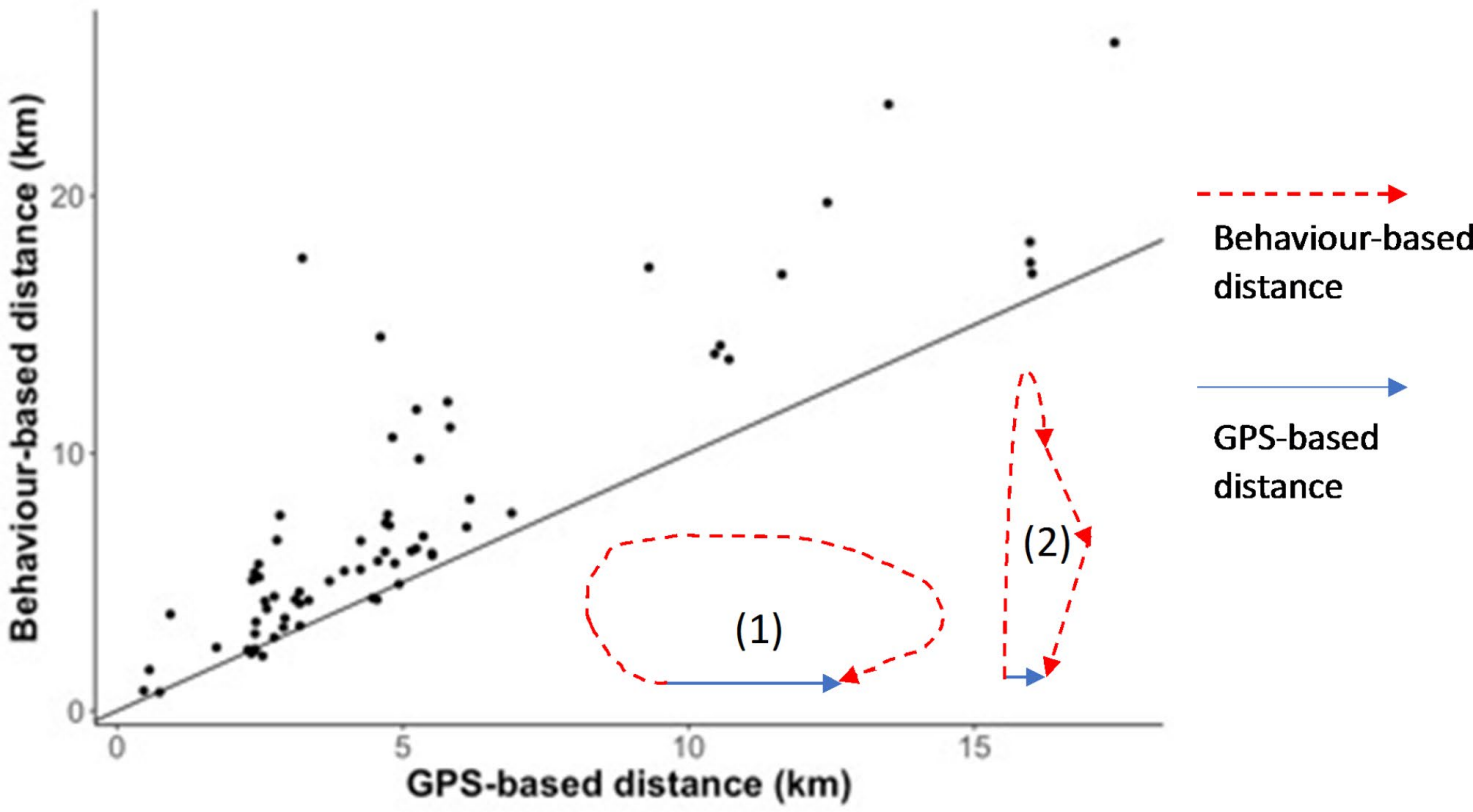
Continuous behaviour records and GPS fixes of duck 5210 from 21/11/2020 to 31/01/2021 (i.e. 68 days, excluding 4 days with failed fixes) for daily distance estimation. Scatterplot showing that the daily distances travelled based on continuous behavioural records are significantly larger than distances calculated from hourly GPS fixes (mean GPS-based distance = 5183 m, mean behaviour-based distance = 7665 m). The diagonal represents a 1:1 ratio. Insets [1] and [2] are schematic scenarios for two GPS fixes (i.e. with one hour difference in this study), where blue lines indicate GPS-based distances (i.e. Haversine distance between two fixes) and red dotted lines indicate possible true flight routes if behaviour-based distance is larger than GPS-based distance

The daily behaviour-based travel distances of d5210 were significantly larger than GPS-based distances (Fig. 5; paired t-test, mean difference = 2482 m, where mean GPS-based distance = 5183 m; t = -7.154, P < .001). Two non-exclusive explanations exist for this discrepancy. Firstly, the animal may not exclusively fly along a straight-line between consecutive fixes (Fig. 5 − 1). Secondly, the animal might have multiple separate flights between two adjacent GPS locations (Fig. 5 − 2).

**Fig. 6:**
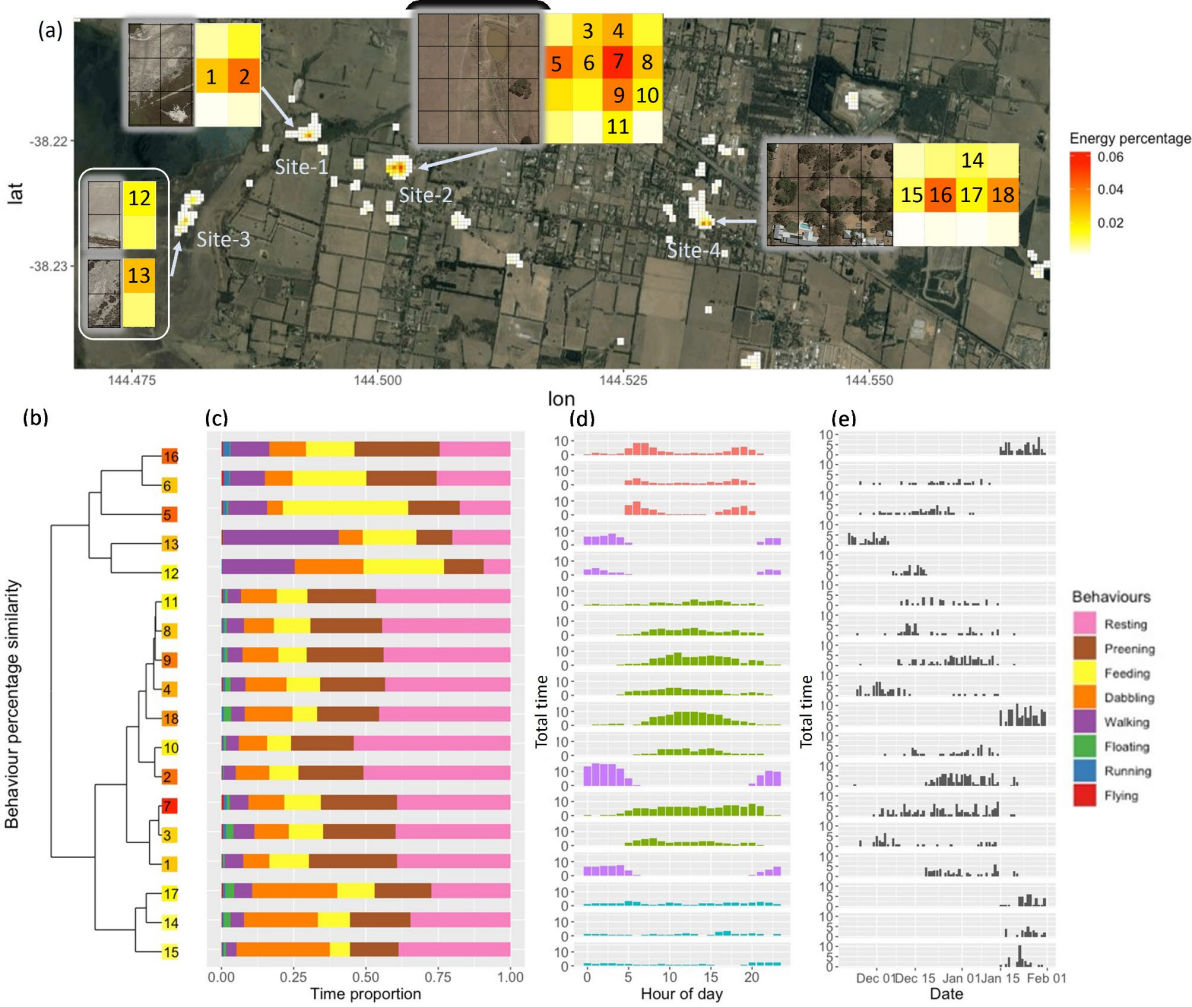
Home range estimation of duck 5210 based on energy expenditure approximation, behaviours and geographical and temporal information. (a). Map depicting duck 5210’s whereabouts between 21/11/2020 and 31/01/2021 (i.e. 72 days). A total of 18, 30 × 30 m cells on this map could be identified in which duck 5210 spent more than 24 h individually. Based on geographical proximity, these 18 cells could be allocated to four key sites (cells 12 and 13 belong to one site). The four insets depict enlargements of these 18 cells. Within each inset, the base map is on the left and on the right the colour of the number in each cell identifying the site and the background colour the energy percentage (see text). (b). Hierarchical clustering of the 18 cells based on (c) the behaviour (i.e. proportional distribution of behaviour) of duck 5210 in each cell. (d). Total time (over the 72 days) in each cell as a function of time of day. Colors of histograms represent hierarchical clustering based on hour percentage. (e). Total time in each cell as a function of date

The total area occupied by d5210 over the 72 day period was 0.188 km^2^ (by summing the areas of all occupied grid cells in Fig. 6a). Using the behaviour-based approach as advocated by Powell and Mitchell [21] (see Methods), 18 30 × 30 m cells where the animal spent most of its time could be identified forming four geographical clusters (cells 1–2, 3–11, 12–13, and 14–18; Fig. 6a). Notably, however, clustering these 18 cells based on the behaviours expressed in these cells yielded different clusters from the geographical clusters (Fig. 6b). Cells 16, 6, 5, 13 and 12 had larger proportions of walking and feeding behaviours compared to all other cells in which resting and preening were the dominant behaviours (Fig. 6c). Within these important feeding cells for d5210, cells 13 and 12 had larger walking proportions than cells 16, 6 and 5, which might indicate that the food in these two cells was more spread out. Next, clustering the cells based on when they were used during the day resulted in four clusters that were again different from the geographical and behaviour-based clusters (Fig. 6d), showing clear diurnal patterns of cell usage. There was also a seasonal pattern visible (Fig. 6e) with a change in site use with date. For example, in December of 2020 and early January of 2021, d5210 mainly used site-2 as diurnal site, whereas after early January of 2021, d5210 mainly used site-4 as the diurnal site.

## Discussion

In this study, we explored how continuous, on-board recorded behaviours may be used to supplement or improve on results using tracking methods that use no behavioural data or intermittent ACC data acquisition only. We showed that with increasing ACC data sampling interval, the error ratios of daily time-activity budgets estimation increased. This was particularly noticeable in rare behaviours. Time-activity budgets can also be used for activity-based energy expenditure estimation (e.g. [25]). Consequently, continuously recorded behaviours can not only improve time but also energy budget estimation. Also, behaviour-based daily distance estimation was shown to significantly improve distance estimation using GPS fixes only. Finally, in line with Powell and Mitchell’s (2012) notion, the continuously recorded behaviours allow more detailed investigation in how animals use their environment and therewith add an additional dimension to home range estimation.

We illustrated how sampling in bursts rather than continuous recording of ACC data impacts time-activity budget accuracy. Obviously, error ratio increased with increasing sampling interval (Fig. 2A) and the error ratios of rare behaviours (such as flying and running) were larger than those for common behaviours (such as preening and resting). For instance, at a 10-minute sample interval, the error ratios of flying and running were already close to 1 (Fig. 3). With even larger sampling intervals, i.e. from 20 to 60 min, the medians of rare behaviours (i.e. flying, running and floating) are shifting away from zero towards larger positive values (Fig. 2A), which indicates that with longer sampling intervals rare behaviours will be more frequently missed. The expected error ratio estimations assuming independence of behavioural observations (Eq. 2) compared reasonably well with the empirical error ratios from the ducks. However, as evidenced by the dependency estimates (Fig. 4), the assumption that consecutive behaviour records are independent was not met at short sampling intervals. As a general result we found that the error ratio estimations from the duck data (Fig. 3) are slightly lower than the expected error ratios calculated using Eq. (2). To reduce the error ratio the sampling interval needs to be shortened. For studies unable to record behaviour continuously, we suggest using Eq. 2 to gauge the most appropriate sampling interval in view of the expected proportions in which the behaviours of interest are likely to be shown. Next, researchers could also consider to have daily variations in sampling interval (e.g. daytime and night-time settings) depending on when focal behaviours are expected to be displayed. Although we here investigated statistical error due to sampling rate in the framework of biologging, similar attention should probably also be given to studies using more conventional behaviour-observation methods [26].

Aside from obtaining more accurate time-activity budgets (i.e. durations of behaviours), the continuous recordings also allowed accurate assessment of the frequencies and sequences of the behaviours. We used flying behaviour as an example to illustrate how such increased accuracy can assist with informing us on details of the animals’ life. We showed behaviour-based daily distance was up to 540% larger than GPS-based daily distance, which is consistent with the findings of Rowcliffe, Carbone [22] and Magowan, Maguire [27].

To illustrate how continuous behavioural monitoring when animals are out of sight opens up new avenues for detailing animals’ environmental requirements and status, following the idea of Powell and Mitchell [21], we assessed the behaviour-based home range of one duck over a period of 72 days. Over this period the individual duck appeared to have a rather consistent daily routine using four sites consisting of respectively 2, 9, 2 and 5 raster cells of 30 × 30 m each (Fig. 6a). However, the behaviours of the duck expressed in these four different sites (and cells therein) varied greatly (Fig. 6b and c). There were for instance just 5 cells (i.e. 16, 6, 5, 13, 12) in which feeding was concentrated (Fig. 6b). Also, the pattern of site use showed a clear diurnal pattern, with sites 1 and 3 being used during the night, and sites 2 and 4 during the day (Fig. 6d). However, although sites 1 and 3 were both used as nocturnal sites, they were used differently. At site-3, the duck spent more time feeding and walking compared to site-1. Diurnal sites 2 and 4 were functionally similar, each site having a very small 2 and 1 cell area in which feeding was concentrated, the remaining area within the sites being used for resting mainly. To understand why there was a seasonal shift of the duck from site-2 to site-4 in mid-January 2021 (Fig. 6e), we surveyed the local environment of site-2, where cell 5 and part of cell 6 appeared to be oat fields that were harvested in early January, explaining the behavioural change. The fine scale use of the landscape through continuous behaviour records as shown in this study can provide pinpoint guidance for the effective and accurate investigation of an animal’s interaction with the landscape, with behaviour-based home ranges potentially providing valuable guidance for conservation.

Understanding of an animal’s behaviour is important in animal studies and various models have been employed to evaluate an animal’s behavioural state from movement data. Models employed to this effect include for instance state-space models (SSM), hidden Markov models and behavioural change-point analyses (e.g. [28–30]). Adding continuous behaviour records to positional data could importantly supplement the results of these models or possibly also replace them; instead of using statistical models to infer behavioural states, continuous behaviours classified through ACC data likely result in finer resolution and more detailed behaviour information.

Although in this study we focussed on exploring how on-board continuously recorded behaviours can improve previous methods, there is great potentials for other useful ACC derived metrics to be calculated on-board and provide valuable data for different research. The trackers used in this study already calculated a default ACC based ODBA index that was stored every 10-minutes. The favourable battery efficiency of this default feature indicates that other continuous on-board calculation and storage of ACC based indices are a possibility. Also, for biomechanics studies, frequency components [31] derived from ACC data could potentially be extracted, stored and transmitted at regular intervals. For reliable extraction of frequency components, one should follow the Nyquist-Shannon criterion as also suggested in [17, 32]. In addition, the field of machine learning is continuously developing and novel computational solutions might also offer promising alternatives, such as the tiny machine learning technology (tinyML; e.g. [33]). Also, on-board application of unsupervised machine learning models should be possible and should be considered since behavioural observations in free-ranging individuals might be difficult to obtain in some animal species.

## Conclusion

We showed that by using trackers allowing for continuous recording of animal behaviour, substantial improvements in the estimation of time-activity budgets and daily traveling distances can be made. With integrating behaviour into home-range estimation we also highlight that this novel tracking technique may not only improve estimations but also open new avenues in animal behaviour research, importantly improving our knowledge of an animal’s state while it is roaming the landscape. Big-data approaches and high-throughput wildlife tracking are opening new research frontiers in biology and ecology [32]. We hope the new insights discussed in this study can trigger more method developments and applications using high-throughput animal behaviour records.

## Electronic supplementary material

Below is the link to the electronic supplementary material.

Supplementary Material 1

## Data Availability

The datasets analysed during the current study are deposited at https://doi.org/10.6084/m9.figshare.21360777.v1.

## List of abbreviations

**ACC**: Accelerometer
**ODBA**: Overall Dynamic Body Acceleration
**VeDBA**: Vectorial Dynamic Body Acceleration
**RMS**: Root Mean Square
**tinyML**: tiny Machine Learning
