# Supplementary material for: Increasingly detailed insights in animal behaviours using continuous on-board processing of accelerometer data
Source: Mov Ecol. 2022 Oct 24;10:42. doi: 10.1186/s40462-022-00341-6 (PMC9594961; doi:10.1186/s40462-022-00341-6)
Supplement: Supplementary file 1 — Supplementary Material 1 [file 40462_2022_341_MOESM1_ESM.pdf]

Supplementary Material

Abstract

Calculation of error ratios and measures of dependence for rare and common behaviours as a function of sampling interval length.

1 The data

Behavioural data of animals is categorized into k categories. By $p_{h,1}, \dots, p_{h,k}$ we will denote the fraction of time animal h, $h = 1, \dots, H$, spends in category $1, \dots, k$, respectively. So, $p_{h,j}$ is the probability animal h shows behaviour from category j when observed at a random point in time. This means we are dealing with a multinomial distribution with parameters 1 and $p_{h,1}, \dots, p_{h,k}$, $\mathrm{Mult}(1, p_{h,1}, \dots, p_{h,k})$, for this one observation.

Behavioural data is scored at interval lengths $\ell_i,\ i = 1, \dots, L$. Here we assume there are no dependencies in each of these discretized time series; however, see Sections 3 and 4 below.

This implies that for animal h at interval length ℓ_i we have a total of $n_{h,i}$, say, independent observations from the $\mathrm{Mult}(1, p_1, \dots, p_k)$ distribution, and that we have to deal with a $\mathrm{Mult}(n_{h,i}, p_{h,1}, \dots, p_{h,k})$ distribution. Observe the smaller the length ℓ_i the larger the sample size $n_{h,i}$, i.e.,

$$\ell_i n_{h,i} = c_h \tag{1.1}$$

holds for some constant c_h depending on the total observation time of animal h.

To formulate it differently, the data for animal h at interval length ℓ_i constitute a realization of a random vector $(X_{h,i,1}, \dots, X_{h,i,k})$ with distribution $\mathrm{Mult}(n_{h,i}, p_{h,1}, \dots, p_{h,k})$. Note that $X_{h,i,j}$ has a binomial distribution with parameters $n_{h,i}$ and $p_{h,i,j}$ and that hence the coefficient of variation for the jth category equals

$$\frac{\sqrt{p_j(1-p_j)}}{p_j} = \sqrt{\frac{1-p_j}{p_j}} = \sqrt{\frac{1}{p_j} - 1}. \tag{1.2}$$

2 The influence of the interval length

We are interested in the size of the standard deviation in estimating $p_{h,i,j}$ relative to the value of $p_{h,i,j}$, in particular in how this error ratio depends on ℓ_i.

Now $p_{h,i,j}$ is estimated by $X_{h,i,j}/n_{h,i}$ and according to Section 1 this estimator has standard deviation

$$\sqrt{\frac{p_{h,i,j}(1-p_{h,i,j})}{n_{h,i}}}. \tag{2.3}$$

Consequently the error ratio equals (cf. (1.1))

$$r_{h,i,j} = \frac{\sqrt{\frac{p_{h,i,j}(1-p_{h,i,j})}{n_{h,i}}}}{p_{h,i,j}} = \frac{1}{\sqrt{n_{h,i}}}\sqrt{\frac{1}{p_{h,i,j}} - 1} = \frac{1}{\sqrt{c_h}}\sqrt{\frac{1}{p_{h,i,j}} - 1}\ \sqrt{\ell_i} \tag{2.4}$$

and hence

$$\ln r_{h,i,j} = -\tfrac{1}{2}\ln c_h + \tfrac{1}{2}\ln \ell_i - \tfrac{1}{2}\ln p_{h,i,j} + \tfrac{1}{2}\ln(1 - p_{h,i,j}). \tag{2.5}$$

This means that the error ratio is a square root function of the interval length as visible in Figure 3; note also that the regression mentioned in the caption of Figure 3 basically resembles (2.5).

The error ratio can be estimated using (cf. (2.4))

$$R_{h,i,j} = \frac{\sqrt{\frac{X_{h,i,j}/n_{h,i}(1-X_{h,i,j}/n_{h,i})}{n_{h,i}}}}{X_{h,i,j}/n_{h,i}} = \frac{1}{\sqrt{c_h}}\sqrt{\frac{1}{X_{h,i,j}/n_{h,i}} - 1}\,\sqrt{\ell_i}. \tag{2.6}$$

Note that this estimator might take the value infinity, since $X_{h,i,j} = 0$ has positive probability.

3 Markov Chains

Consider observations of animal h taken at interval length ℓ_i. A natural model for the behaviour of animals is that the present behaviour has some influence on the behaviour in the immediate future, more precisely, the probability $p_{g,j}$ that the behaviour at time $t + \ell_i$ is j, given the behaviour at time t is g, might deviate from p_j. This leads to a Markov chain as model for the consecutive observations. In our case we have k behaviours (or states in the Markov Chain terminology). The probabilities $p_{g,j},\ g = 1, \dots, k,\ j = 1, \dots, k$, can be arranged in a $k \times k$-matrix (the transition matrix) in which the gth row contains the probabilities $p_{g,j},\ j = 1, \dots, k$. In case of independence between consecutive points in time all rows in the matrix will be the same and equal $(p_1, \dots, p_k)$. Note that the continuous observations yield a very accurate estimate of $(p_1, \dots, p_k)$.

To test if the gth row of the transition matrix equals $(p_1, \dots, p_k)$ one often applies the Pearson χ^2 test; see e.g. $https://en.wikipedia.org/wiki/Chi-squared\_test$. Let n_g be the number of observations (of animal h at interval length ℓ_i) at which the animal shows behaviour g (or is in state g). Let $X_{g,j}$ be the number of times the behaviour changes from g to j; so, $\sum_{j=1}^k X_{g,j} = n_g$. Now the Pearson χ^2 test statistic equals

$$T_g = \sum_{j=1}^k \frac{n_g\left(X_{g,j}/n_g - p_j\right)^2}{p_j} = \sum_{j=1}^k \frac{\left(X_{g,j} - n_g p_j\right)^2}{n_g p_j}. \tag{3.7}$$

Under the null hypothesis of independence, i.e., the hypothesis that all rows of the transition matrix are the same, the test statistic T_g has approximately a χ^2 distribution with $k-1$ degrees of freedom. The fact that n_g is a random variable doesn't matter here. Given n_g the random variable $X_{g,j}$ has a binomial distribution with parameters n_g and p_j.

Since we want to test if all rows of the transition matrix are the same, it makes sense to consider the test statistic

$$T = \sum_{g=1}^k T_g = \sum_{g=1}^k \sum_{j=1}^k \frac{\left(X_{g,j} - n_g p_j\right)^2}{n_g p_j}, \tag{3.8}$$

which has approximately a χ^2 distribution with $k(k-1)$ degrees of freedom under the null hypothesis for reasonably large values of n_g.

4 A measure of dependence

Even the slightest deviation from independence causes the test based on T to reject independence, since we have very many observations. Therefore it makes more sense to consider a measure for the dependence. To this end we fix the interval length ℓ_i and restrict attention to the gth row of the transition matrix, which

we don't know, but which is estimated by $(X_{g,1}/n_g, \ldots, X_{g,k}/n_g) = (q_1, \ldots, q_k)$. In case of independence this k-vector should be close to the k-vector $(p_1, \ldots, p_k)$.

Now, it seems natural to look for a suitable distance measure to determine the distance between these two k-vectors, $p = (p_1, \ldots, p_k)$ and $q = (q_1, \ldots, q_k)$. Note that p and q are elements of the so-called $(k-1)$-simplex, which is the collection of k-vectors with nonnegative components that add up to 1.

A simple distance is the L_1-distance, which has many exotic names; see *https://en.wikipedia.org/wiki/Taxicab_geometry*. It is defined by

$$d_1(p, q) = \sum_{i=1}^{k} |q_i - p_i|, \tag{4.9}$$

whereas the standard Euclidean or L_2-distance is defined by

$$d_2(p, q) = \sqrt{\sum_{i=1}^{k} (q_i - p_i)^2}. \tag{4.10}$$

Let $p_{\min}$ be the minimum value among $p_1, \ldots, p_k$. Then one can show

$$0 \leq d_1(p, q) \leq 2\,(1 - p_{\min})\,. \tag{4.11}$$

Consequently it seems natural to choose as a dependence measure (for each row g and each ℓ_i)

$$\Delta_1(p, q) = \frac{d_1(p, q)}{2\,(1 - p_{\min})} = \frac{\sum_{i=1}^{k} |q_i - p_i|}{2\,(1 - p_{\min})}, \tag{4.12}$$

since the value of $\Delta_1(p, q)$ is in between 0 and 1. Here 0 corresponds with independence in the Markov Chain when starting from state/behaviour g and 1 corresponds with the worst possible dependence.

An important issue is, what values of $\Delta_1(p, q)$ are close enough to 0 to make results based on monitoring with interval length ℓ_i sufficiently reliable.

Proof of (4.11)

Since the simplex Δ^{k-1} is a convex and compact set and since $q \mapsto d_1(p, q)$ is a convex function, this function attains its maximum at an extreme point of the simplex, according to Bauer's maximum principle. These extreme points are the unit vectors. This implies (with e_j the jth unit vector)

$$\max_{q \in \Delta^{k-1}} d_1(p, q) = \max_{j=1,\ldots,k} d_1(p, e_j) = \max_{j=1,\ldots,k} 1 - p_j + \sum_{i \neq j} p_i = \max_{j=1,\ldots,k} 2(1 - p_j) = 2\,(1 - p_{\min})\,. \tag{4.13}$$

$\square$
